# Machine learning techniques for identifying mental health risk factor associated with schoolchildren cognitive ability living in politically violent environments

**DOI:** 10.3389/fpsyt.2023.1071622

**Published:** 2023-05-26

**Authors:** Radwan Qasrawi, Stephanny Vicuna Polo, Rami Abu Khader, Diala Abu Al-Halawa, Sameh Hallaq, Nael Abu Halaweh, Ziad Abdeen

**Affiliations:** ^1^Department of Computer Sciences, Al-Quds University, Jerusalem, Palestine; ^2^Department of Computer Engineering, Istinye University, Istanbul, Türkiye; ^3^Al-Quds Center for Business Innovation and Entrepreneurship, Al-Quds University, Jerusalem, Palestine; ^4^Faculty of Medicine, Al-Quds University, Jerusalem, Palestine; ^5^Al-Quds Bard College for Arts and Sciences, Al-Quds University, Jerusalem, Palestine

**Keywords:** mental health, cognitive abilities, machine learning, prediction, health, social support, nutrition

## Abstract

**Introduction:**

Mental health and cognitive development are critical aspects of a child’s overall well-being; they can be particularly challenging for children living in politically violent environments. Children in conflict areas face a range of stressors, including exposure to violence, insecurity, and displacement, which can have a profound impact on their mental health and cognitive development.

**Methods:**

This study examines the impact of living in politically violent environments on the mental health and cognitive development of children. The analysis was conducted using machine learning techniques on the 2014 health behavior school children dataset, consisting of 6373 schoolchildren aged 10–15 from public and United Nations Relief and Works Agency schools in Palestine. The dataset included 31 features related to socioeconomic characteristics, lifestyle, mental health, exposure to political violence, social support, and cognitive ability. The data was balanced and weighted by gender and age.

**Results:**

This study examines the impact of living in politically violent environments on the mental health and cognitive development of children. The analysis was conducted using machine learning techniques on the 2014 health behavior school children dataset, consisting of 6373 schoolchildren aged 10-15 from public and United Nations Relief and Works Agency schools in Palestine. The dataset included 31 features related to socioeconomic characteristics, lifestyle, mental health, exposure to political violence, social support, and cognitive ability. The data was balanced and weighted by gender and age.

**Discussion:**

The findings can inform evidence-based strategies for preventing and mitigating the detrimental effects of political violence on individuals and communities, highlighting the importance of addressing the needs of children in conflict-affected areas and the potential of using technology to improve their well-being.

## 1. Introduction

The normal cognitive development of children living in conflict areas is crucial, given the adverse situation they face, which can have a long-lasting impact on their well-being. Minimizing the exposure to and impact of other risk factors is important, as research has shown that there is a relationship between cognitive development and future ill mental health. Several studies have highlighted the negative effects of exposure to violence and mental health difficulties on children’s cognitive development (1–4). Mental health disorders, such as depression, anxiety, and stress, have been linked with decreased cognitive functioning, whereas political violence can have a significant impact on the mental health and cognitive ability of children. Exposure to violence and traumatic events, such as war and civil conflict, can result in mental health disorders such as post-traumatic stress disorder (PTSD), depression, anxiety, and stress (1, 2). These disorders can have negative impacts on children’s cognitive development, including their memory, attention, and executive function (4). Moreover, mental health and cognitive ability are two important aspects of human functioning that are closely related and can influence each other. Mental health problems can affect cognitive abilities, while cognitive abilities can also play a role in the development and maintenance of mental health (5, 6).

Several studies have investigated the association between mental health symptoms and cognitive ability and have demonstrated a complex interplay between the two (4–7). Depression has been found to impact attention, memory, and executive function (8). Individuals with depression have been found to perform poorly on tasks requiring sustained attention and memory recall. Moreover, depression has also been found to impair cognitive functions, and impact working memory, which is a crucial component of executive function (9, 10). Additionally, anxiety has also been linked with decreased cognitive functioning, particularly in the areas of attention and memory. Several studies assessed the relationship between anxiety and cognitive ability (1, 11, 12). Findings show that children and youth with mental health symptoms were associated with low cognitive functions, such as children with social fears, who showed specific types of memory deficit, and children with social problems may have neurodevelopmental delays compared to other children (11). Further studies evidenced that children with greater anxiety symptoms are more likely to have difficulties in cognitive activities, such as problem-solving (11, 13). Furthermore, stress has also been found to have negative effects on cognitive functioning. Chronic stress has been linked with decreased performance in tasks involving memory and executive function (14–16). Particularly, stress has been found to impact working memory, which is a crucial component of executive function (17). Stress has also been found to impact attention and memory recall, particularly when the information being remembered is emotionally charged (15).

Children living in political violence and conflict environments were found to be subject to mental health problems that consequently affect their cognitive skills. Children who are exposed to political violence often experience high levels of stress and trauma, which can affect their ability to form healthy attachments and relationships (1, 2, 4). This, in turn, can negatively impact their cognitive and social development. Additionally, children who are exposed to political violence are more likely to experience disrupted sleep patterns, which can have negative impacts on their cognitive abilities, including attention and memory (18).

In recent years, machine learning (ML) has been increasingly used as a tool for identifying and understanding the associated factors affecting cognitive ability, such as mental health, sociodemographic characteristics, lifestyle, and political violence (19–23). Several studies have utilized ML algorithms, such as decision trees, random forests, and support vector machine algorithms, to identify the impact of political violence on children’s mental health outcomes, such as PTSD and depression (24, 25). These studies have found that ML models can accurately identify children’s mental health outcomes based on factors such as exposure to violence, trauma history, and demographic characteristics (26). Moreover, ML has been used in estimating mental health issues, and to explore the potential benefits and challenges associated with this approach, including the ability to analyze large amounts of data from multiple sources, improving the accuracy of identifications, and its cost-effectiveness (21, 26–28).

To the best of our knowledge, this is the first study that used different ML techniques to identify the associated risk factors with children’s cognitive ability living in a conflict area. The study compared ML techniques and identified the most important factors that affect cognitive ability. Furthermore, the study produced a ML model that could be used in clinical and educational applications for improving cognitive ability and mental health identification among schoolchildren.

## 2. Materials and methods

### 2.1. Dataset

The dataset used in this study consists of a sample of 6,374 schoolchildren, and 31 associated features. The dataset consists of primary data extracted from the national Health Behavior in School-Aged Children (HBSC) study [derived from the international HBSC study (29)] conducted in the Palestinian territories by Al-Quds University and the Ministry of Education in the academic year 2013–2014. The data set was weighted and adjusted by gender (50% Boys, and 50% Girls) and grade, including students in grades 5th, 6th,7th, 8th, and 9th (19.9%, 20.2%, 21.1%, 19.3%, and 19.5%, respectively), within the ages of 10–15 years.

The data type is a mix of numerical and categorical variables. Numerical variables include the children’s age, cognitive score, and academic performance, while categorical variables include sociodemographic, mental health, political violence, physical health, and lifestyle variables. To prepare the data for analysis, the interquartile range (IQR) method was used to identify the outliers, and manual inspection and data cleansing methods were used to identify the incorrect data entry values, while the missing values were input using the median imputation method, and irrelevant features were removed through a feature selection process, in which we used the correlation analysis, mutual information techniques to identify the most relevant and informative features.

The labels of the target variables indicated whether children had low, average, or above-average cognitive scores. The random under sampling technique was used to balance the class distribution with 3,187 samples for each class. The classes were balanced to have unbiased, more accurate and to ensure fairness in models’ identification, The data was split into training sets for learning (70%), testing (20%), and validation (10%). Evaluation metrics used in this study include accuracy, F1 score, and receiver operating characteristic (ROC) curve analysis. These metrics were chosen to provide a comprehensive evaluation of the model’s ability to correctly classify schoolchildren’s cognitive abilities. The dataset is published on DANS EASY open access database: https://doi.org/10.17026/dans-zzt-guh7.

### 2.2. Study variables

The ML model features were listed in Table 1, including variable names, values, and levels of classification. The balanced cognitive score was used as the study target variable.

**Table 1:** List of machine learning model variables.

Variable	Description
Cognitive_Ability	Below average, average, and above
Gender	Boys, Girls
Age (years)	10–11, 12–13, 14–15
Living_Place	Urban, Rural, Camp
Body Mass Index	Underweight, normal, overweight, and obese
Father_Education	≤Secondary, >Secondary
Mother_Education	≤Secondary, >Secondary
School_type	Public, UNRWA
Family_Income	Low, Moderate, High
Physical_Activity	Low, Moderate, High
Leisure_Time_Activity	Low, Moderate, High
Smoking_Tobacco	Yes, No
Healthy_Food_Consumption	Yes, No
Depression_Symptoms	Normal: 0–11, Depressed: ≥12
Anxiety_Symptoms	(Low: 0–9, Moderate: 10–14, and High:15–21)
Mental_Health_Difficulties	Low: 0–14, Moderate: 15–17, and High:18–40
Post-traumatic_stress disorder	0 = No PTSD, 1 = Moderate PTSD, and 3 = Severe
PsychosomaticSymptom	Yes, No
Exposure_Political Violence	No Exposure, Moderate, and Severe
Child_Maltreatment	Never, Some and Severe
Sleeping_Hours	≥8 h per day, <8 h per day
Family_Support	Low, Moderate, High
Peer_Support	Low, Moderate, High
School_Support	Low, Moderate, High
Positive_Health_Perception	Positive, Negative
Life_Satisfaction	Satisfied, Unsatisfied
Facing_School_Violence	Low, Moderate, High
Child_Abuse	Low, Moderate, High
Bullying	Never, Mild, High
Academic_Performance	Low, Moderate, High
Suicide_Attempt	Yes, No

#### 2.2.1. Sociodemographic variables

The sociodemographic variables describe the social and demographic characteristics of the study participants, including age, parents’ education, family income, place of residence, school type, and Body Mass Index (BMI) [BMI = weight in kg/(height in m^2^)].

#### 2.2.2. Lifestyle

The lifestyle variables describe the children’s behaviors and habits that might impact their overall health and well-being. In this study, physical activity, leisure time activity, smoking, sleeping, and food consumption were included. The physical activity and leisure time activities were measured by collecting data on the rate of physical exercises over 60 min and categorized according to the WHO definition [low (>3 days per week), Moderate (3–5 days per week), and High (6–7 days per week)], while the leisure time (screen time) activity was categorized into [low (>2 h per day), moderate (2–3 h per day), and high (≥4 h per day)] (30). The smoking variable was categorized into “smoker and non-smoker,” the sleeping variable was categorized into whether subject sleeps on average less than or equal to 8 h/day, or more than 8 h/day (≥8 h/day, or <8 h/day), and the food consumption was categorized into “healthy” based on the consumption rate of vegetables, fruits, milk or yogurt, and dairy products, or “unhealthy” based on the rate of consumption of soft drinks, energy drink, sweets, and sugar, both on a weekly basis.

#### 2.2.3. Mental health symptoms

The mental health variables include depression, anxiety, stress, psychosomatic, and posttraumatic stress disorder (PTSD). The depression symptoms were measured by the 18-item Birleson Depression Self-Rating Scale for Children (DSRS), which was calculated by summing the items’ answers and categorizing them into two categories (Normal: 0–11, Depressed: ≥12) (31). The anxiety levels were measured using the General Anxiety Disorder-7 (GAD-7) scale, which was calculated by summing the items’ answers and categorizing them into three categories (Low: 0–9, Moderate: 10–14, and High:15–21) (32). Overall emotional and behavioral problems among children were measured using the Strengths, and Difficulties Questionnaire (SDQ) scale, which was categorized into three groups (Low: 0–14, Moderate: 15–17, and High:18–40) (33).

The psychosomatic symptoms were assessed using an 8-item psychosomatic symptoms scale (Cronbach’s alpha = 0.85), in which children were asked if they experience the following symptoms at least once a week: headache, stomachache, backache, or dizziness. They were further asked if they experienced the following symptoms at least once a day: feeling depressed, irritability or bad temper, feeling nervous, difficulties in getting to sleep, and/or feeling dizzy. Participants answered on a scale from 1 (every day) to 5 (rarely or never). The answers were grouped into a continuous variable, which was categorized into a dichotomous variable [1: occurrence of the symptom (every day or more than once a week), and 0: no symptoms (Once a week, once a month, or never)] (34).

Posttraumatic Stress Disorder (PTSD) was measured by the 20 items index scale used by the HBSC survey, which determines the level of posttraumatic stress severity among children (34). The scale is composed of a 5-point scale from 0- “not at all” to “very much.” The PTSD level was measured by categorizing the total score into three groups: 0 = No PTSD, 1 = Moderate PTSD, and 3 = Severe PTSD (34).

#### 2.2.4. Political violence

Children’s exposure to political violence was measured using the political violence inventory scale regarding children’s exposure to military violence designed by Haj-Yahia et al. (35). The scale is composed of 40 statements that measure three levels of exposure: (1) very severe exposure (Personal or family member injured or hurt by military incursion), (2) moderate exposure (present at military incursion or seeing someone hurt or injured by military attack), and (3) no exposure (no direct contact with military incursion) (36).

#### 2.2.5. Maltreatment

Child maltreatment measures any act or series of acts of bad treatment by parents or family members that results in harming the children. The scale is composed of 8 items (Cronbach’s alpha = 0.87) that measure physical abuse, sexual abuse, neglect, and exposure to domestic violence. Participants responded on a scale of (Very True, True, or Not True), the scale was categorized into Never: (21–24 that includes participants who responded to not true to most or all of the items), Moderate: (16–20 that includes participants who responded true to all or most of the items) and Severe score: (8–15 that includes participants who responded to very true to all or most of the items) (18).

#### 2.2.6. Social support

Social support measures the relationship and the help that children receive from their parents, friends, and school. Social support is measured by three subscales, each subscale is composed of a list of items that include (1) “family help,” “emotional help,” “ability to talk,” and “help in making decisions,” (2) “Friends try to help,” “can count on friends,” “having friends to share the joy with,” and “can talk to friends about problems,” and (3) “Teacher accepts me,” “teacher cares about me,” and “feel trust in teacher.” These items were summed and categorized into three groups: Low (0–12), Moderate (13–16), and High levels of support (17–24) (34).

#### 2.2.7. Positive health perceptions

The Positive Health Perception Scale (PHPS) to assess an individual’s perception of their own health. It was designed to measure positive health perceptions, including attitudes, beliefs, and values related to health. The students were asked to rate their agreement with each statement on a Likert scale, that ranged from strongly disagree to strongly agree. The scale total score was classified into two groups: The scores of 35 or above was used to indicate a positive perception of health, while a score below 35 was considered a negative perception of health (34).

#### 2.2.8. Life satisfaction

The Cantril Ladder satisfaction scale was used to measure children’s life satisfaction. The scale ranged from 0 to 10, where 10 is the best possible life and 0 is the worst possible life (37). The scale was classified according to Mazur et al. in which the scale was classified into: Low (0–6), average (7–8), and high (9–10). While in this study, the ML model was designed to focus on the low level of satisfactions, so we regrouped the responses into: Unsatisfied (0–6) and Satisfied (7–10) (38).

#### 2.2.9. School violence

Violence was measured by asking children if they were involved in physical fights; carrying weapons such as solid objects, knives, or other objects; how many times they were injured and treated by physical fights; or if they were involved in bullying other students. The scale is divided into two groups: Low (0–2); and High level (3–4). Higher scores indicated higher levels of violence.

#### 2.2.10. Academic performance

The student’s academic performance was measured based on the students’ average grades score; the grades were collected from the school grading system. The Grade Point Average (GPA) score was used for classifying the total grades into three groups: Low: ≤59; Moderate 60–79; and High ≥80.

#### 2.2.11. Cognitive abilities

Students’ cognitive ability scores were assessed through the Cognitive Abilities Test (CogAT) is a standardized test used to measure cognitive abilities in students from kindergarten through grade 12 (or grade 13 in some regions). The test assesses students’ abilities in three areas: verbal, quantitative, and nonverbal reasoning. The verbal reasoning section of the CogAT assesses a student’s ability to use and understand language, including the ability to detect relationships between words, to recognize synonyms and antonyms, and to understand figurative language. The quantitative reasoning section assesses a student’s ability to reason with numbers and to solve mathematical problems, including arithmetic, algebra, and geometry. The nonverbal reasoning section assesses a student’s ability to reason with shapes and images, including the ability to recognize patterns, to complete sequences, and to understand spatial relationships. Each feature had a detailed content item, such as vocabulary, series, analogies, and inference. Overall, 181 content items were used for assessing the students’ intelligence abilities. The total score was estimated from the detailed scores with an average of 60.7 ± 16.7 points (39). The cognitive scores were further classified into two categories: (1) below average, and (2) average and above average, for enhancing the performance of the ML algorithms.

#### 2.2.12. The suicidal ideation and behavior

The HBSC survey designed a scale of 4 items for measuring suicide ideation among school children. The scale measures the severity level of suicidal ideation and behavior. In this study, only the question related to serious thoughts of attempting suicide was considered. The variable is composed of two categories: Yes or No (34).

### 2.3. Machine learning models

The ML models include Gradient Boosting (GB), Support Vector Machine (SVM), Random Forest (RF), Artificial Neural Network (ANN), k-nearest neighbors (k-NN), and Decision Tree (DT) algorithms, these models were built and compared based on their performance measures. The performance of the models was evaluated using a variety of metrics, including accuracy, precision, recall, and F1-score. The models’ features were structured based on the target variable (cognitive ability), and associated factors that include the list of variables as indicated in Table 1. The selected model was trained on a 70% random sample of the data, and the remaining 30% was used for model testing and validation. The parameter optimization was performed using the grid search method and 10-fold cross-validation approach for the used models. The optimal parameters for each model were selected as follows:

1. The ANN model had a hidden layer with 1,000 neurons, a regularization parameter of 0.0001, and a maximum of 600 iterations using the logistic activation function.
2. The Random Forest model had 1,000 trees with a maximum depth of 5, a minimum number of samples at each leaf node set to 1, and a maximum number of samples to split internal nodes set to 2.
3. The SVM model had a regularization parameter of 20, a Radial Basis Function (RBF) kernel with a value of 0.001, and a bias error control factor set to 1.
4. The Gradient Boosting model had 1,000 trees with a learning rate of 0.1 and a maximum depth of 3 for individual trees.
5. The KNN model used 10 nearest neighbors, a uniform weighting function, and the Euclidean distance metric.
6. The Decision Tree model had a maximum tree depth of 100, the number of instances in leaves set to 2, and the smallest subsets set to 5.

Based on the optimized parameters, the algorithms were used to identify cognitive abilities.

### 2.4. Data analysis

Three approaches of data analysis were used to identify the association between cognitive ability and the associated risk factors. Statistical analysis, machine learning analysis and Gini importance analysis.

#### 2.4.1. Statistical analysis

To summarize the demographic characteristics of the study population, we conducted descriptive statistics, which involve the use of summary statistics to describe the central tendency, variability, and distribution of the data. This analysis provided an overview of the characteristics of the study population, including age, gender, and socioeconomic status.

To test the relationship between the study variables and cognitive ability, we used inferential statistics, which involve the use of statistical tests to determine the significance of the relationships between variables. Specifically, we used the binary regression analysis to explore the relationship between variables, including the odd ratio, which is a measure of the strength of the association between the cognitive ability and the independent variables. Additionally, we used analysis of variance (ANOVA) to compare the means of different groups, including the calculation of the *F*-value, which is a measure of the overall significance of the model. Furthermore, the Univariate analysis was also conducted to assess the distribution and relationship of cognitive ability with other variables.

#### 2.4.2. Machine learning analysis

Data preprocessing techniques were conducted prior to the implementation of the ML models, including cleaning, transformation, and normalization processes. The final dataset consisted of 6,374 participants. The six ML models were built and performed using the Python orange data mining software (40), which was used for testing and validating the ML models. The study employed a 10-fold cross-validation approach to evaluate the performance of the machine learning models.

The evaluation of ML models for identifying cognitive ability levels in students and associated risk factors involves assessing the effectiveness and reliability of the models using various performance measures. Some commonly used performance measures include balanced accuracy, specificity, precision, recall, and F-measure (the harmonic mean of precision and recall), in addition to the area under the receiver operating characteristic curve (AUC-ROC), was used to evaluate the performance of binary classifiers. The Wilcoxon signed-rank test is a non-parametric statistical test used to compare the performance of two models on a given data set. In this study, we used the Wilcoxon signed-rank test to determine whether there was a significant difference in performance between two machine learning models and helped us to identify the model that performed better on the given dataset.

#### 2.4.3. Gini importance analysis

We utilized Gini importance analysis to identify the most important risk factors that contribute to low cognitive ability scores among the study population. Gini importance analysis involved calculating the Gini importance coefficient for each potential risk factor, which allowed us to determine the relative importance of each factor in explaining the variation in cognitive ability scores. We used Python Anaconda software to conduct the analysis and generate the results.

#### 2.4.4. Classification and regression trees

In this study, the Classification and Regression Trees (CRT) technique was utilized as a ML approach to identify the patterns of associations between cognitive ability and study variables. The CRT method is a decision tree-based technique that enables the identification of complex nonlinear relationships between predictor variables and an outcome variable. It uses a recursive partitioning algorithm to split the data set into increasingly homogeneous subsets based on the predictor variables’ values. The CRT technique produced a decision tree that provided a visual representation of the complex relationships between cognitive ability and other predictor variables in the study and helped us to better understand the factors that contribute to cognitive ability levels.

## 3. Results

### 3.1. Descriptive analysis

The results in Table 2 showed the descriptive univariant analysis of children’s cognitive ability with sociodemographic variables. The data set was balanced and weighted by gender and age, with equal representation of both boys and girls across age groups. Of the participants, 28.7% reported low cognitive ability scores, with a higher percentage reported by boys (38.8%) compared to girls (18.5%). The prevalence of low cognitive scores varied by age, with an average of 29% across the three age groups (10–11, 12–13, 14–15). In terms of place of residence, urban and camp residents had a higher percentage of low cognitive scores (31.7 and 28.4%, respectively). Additionally, participants with parents who had lower than a secondary school education had a higher percentage of low cognitive scores (31.6% for father’s education and 32.8% for mother’s education). Moreover, public school students had a higher percentage of low cognitive scores compared to UNRWA schools (33.1 and 22.9%, respectively). These results provide important insights into the sociodemographic factors associated with low cognitive ability scores among schoolchildren living in politically violent environments.

**Table 2:** The statistical analysis of Childrens’ cognitive ability scores by sociodemographic variables.

Variable	Feature	Cognitive ability score		*F* (*p*-value)	OR (95% CI)
		Low	Average and above		
		*n* (%)			
Gender	Boys	1,238 (38.8)	1,949 (61.2)	317.3 (0.001)	2.03 (1.76–2.33)
	Girls	591 (18.5)	2,596 (81.5)		
	Total	1,829 (28.7)	4,545 (71.3)		
Children age	10–11	642 (29)	1,571 (71)	2.22 (0.108)	0.89 (0.82–0.97)
	12–13	677 (28)	1,744 (72)		
	14–15	510 (29.3)	1,230 (70.7)		
Place of residence	Urban	909 (31.7)	1,959 (68.3)	20.7 (0.001)	0.87 (0.8–0.95)
	Rural	494 (24.6)	1,511 (75.4)		
	Camp	426 (28.4)	1,075 (71.6)		
Father education	≤Secondary	573 (23.9)	1,826 (76.1)	5.9 (0.015)	0.85 (0.73–0.98)
	>Secondary	1,256 (31.6)	2,719 (68.4)		
Mother education	≤Secondary	488 (21.4)	1,795 (78.6)	25.7 (0.001)	0.81 (0.69–0.94)
	>Secondary	1,341 (32.8)	2,750 (67.2)		
Physical activity	Low	382 (26.5)	1,058 (73.5)	34.1 (0.001)	1.13 (1.05–1.23)
	Moderate	643 (35.9)	1,147 (64.1)		
	High	804 (25.6)	2,340 (74.4)		
Leisure time activity	Low	478 (30)	1,116 (70)	8.2 (0.001)	1.17 (1.08–1.26)
	Moderate	564 (32.2)	1,186 (67.8)		
	High	787 (26)	2,243 (74)		
Family income	Low	802 (29)	1,960 (71)	1.4 (0.241)	1 (0.92–1.09)
	Moderate	688 (29.1)	1,673 (70.9)		
	High	339 (27.1)	912 (72.9)		
School type	GOV^1^	1,200 (33.1)	2,425 (66.9)	145.6 (0.001)	1.88 (1.62–2.18)
	UNRWA^2^	629 (22.9)	2,120 (77.1)		
Body mass index (BMI)	Underweight	84 (25.6)	244 (74.4)	1.1 (0.348)	1.2 (0.83–1.7)
	Normal	1,528 (29.6)	3,641 (70.4)		
	Overweight	138 (23.6)	446 (76.4)		
	Obese	79 (27)	214 (73)		

1 GOV, government.

2 UNRWA, United Nations Relief and Works Agency; OR, Odd ratio; F, Fisher’s exact test; (95% CI), 95% Confidence Interval.

Univariate analysis showed significant associations between cognitive ability and gender, place of residence, father’s education, mother’s education, physical activity, leisure time activity, school type, exposure to political violence, PTSD, depression, SDQ, maltreatment, positive health perception, academic performance, healthy food consumption, tobacco risk, parental support, school violence, and suicidal ideation (*F* values ranging from 4.6 to 317.3, all *p*s < 0.05 except for anxiety, friend support, school support, family income, physical activity, and life satisfaction).

The results in Table 2 showed that there were no significant associations with age, family income, and BMI. The analysis by academic performance showed that participants with below-average academic scores had reported a lower cognitive ability (42%). Moreover, participants who did not consume healthy food on a regular basis, smoke, have low levels of parental support, were exposed to school violence ≥4 times, and thought of attempting suicide reported a higher percentage of low cognitive scores (33.3, 48.1, 32.3, 44.6, and 44%, respectively).

Results in Table 3 showed that 71.1% of participants had moderate or severe exposure to political violence, of which 42.8% had low cognitive ability scores. Participants with moderate or severe PTSD had a higher rate of low cognitive ability (43.7%) than other participants (25.8%). Participants with severe depression reported a higher percentage of low cognitive scores compared to moderate and low depression scores (31, 28.8, and 28.2%, respectively). The measurement of emotional and behavioral problems among children indicated that the participants with no emotional or behavioral problems reported a higher rate of low cognitive scores than other groups (33%). The study further assessed the effect of family maltreatment on children’s cognitive ability, and findings indicated that a high level of maltreatment was associated with a high level of low cognitive scores (52%). Furthermore, the results of the logistic regression analysis showed that academic performance, gender, satisfaction, school type, and exposure to political violence were the most five important factors affecting cognitive abilities among school children.

**Table 3:** The statistical analysis of Childrens’ cognitive ability scores by mental health and political violence factors.

Variable	Feature	Cognitive ability scores		*F* (*p*-value)	OR (95% CI)
		Low	Average and above		
		*n* (%)			
Exposure to political violence	No	860 (46.7)	980 (53.3)	19.1 (0.001)	1.39 (1.2–1.63)
	Moderate	516 (21.4)	1,899 (78.6)		
	Severe	453 (21.4)	1,666 (78.6)		
PTSD	Low	316 (16.3)	1,618 (83.7)	7.7 (0.001)	0.83 (0.75–0.92)
	Moderate	619 (25.8)	1,776 (74.2)		
	High	894 (43.7)	1,151 (56.3)		
Depression scale	Low	668 (28.2)	1,705 (71.8)	4.6 (0.010)	1.19 (1.06–1.34)
	Moderate	1,048 (28.8)	2,588 (71.2)		
	Severe	113 (31)	252 (69)		
Behaviours strengths and difficulties	Normal	797 (33)	1,620 (67)	7.6 (0.001)	1.14 (1.06–1.23)
	Mild	538 (25.1)	1,608 (74.9)		
	Abnormal	494 (27.3)	1,317 (72.7)		
Child maltreatment	No	692 (19.2)	2,910 (80.8)	40.1 (0.001)	0.77 (0.7–0.84)
	Moderate	297 (25.7)	859 (74.3)		
	High	840 (52)	776 (48)		
Positive health	Negative	436 (18.3)	1,946 (81.7)	19.5 (0.001)	0.69 (0.6–0.81)
	Positive	1,393 (34.9)	2,599 (65.1)		
Life satisfaction	Satisfied	1,186 (30.5)	2,703 (69.5)	15.9 (0.001)	2.08 (1.91–2.26)
	Unsatisfied	643 (25.9)	1,842 (74.1)		
Academic performance	Below average	1,338 (42)	1,847 (58)	169.6 (0.001)	2.1 (1.9–2.3)
	Average	295 (19.4)	1,224 (80.6)		
	Above average	187 (11.6)	1,431 (88.4)		
Healthy food consumption	No	573 (22)	2,032 (78)	18.9 (0.001)	0.65 (0.56–0.76)
	Yes	1,256 (33.3)	2,513 (66.7)		
Tobacco risk	No	1,226 (23.9)	3,895 (76.1)	28.9 (0.001)	1.24 (1.13–1.35)
	Yes	603 (48.1)	650 (51.9)		
Parental support	Low	641 (32.3)	1,346 (67.7)	12.3 (0.001)	1.03 (0.94–1.13)
	Moderate	855 (31.5)	1,861 (68.5)		
	High	333 (19.9)	1,338 (80.1)		
School violence	Never	512 (23)	1,710 (77)	14.2 (0.001)	0.91 (0.94–1 0.13)
	1–3 times	684 (25)	2,048 (75)		
	4+ times	633 (44.6)	787 (55.4)		
Seriously thought of attempting suicide	Yes	572 (44)	729 (56)	191.9 (0.001)	0.92 (0.78–1.09)
	No	1,257 (24.8)	3,816 (75.2)		

OR, Odd ratio; F, Fisher’s exact test; (95% CI), 95% Confidence Interval.

#### 3.1.1. Machine learning performance analysis

Several ML models were used to assess the performance of ML techniques in identifying cognitive ability from the associated factors. Results in Table 4 showed the ML models’ performance analysis AUC, Balanced accuracy, F1, recall, and precision. The results indicated that the RF had the highest performance of balanced accuracy (87%), followed by NN and SVM. While the lowest balanced accuracy rate was found in the KNN algorithm. In terms of execution time, KNN, Decision Tree and RF reported the lowest execution time (0.001 s, 0.03 s, and 0.62 s). However, all ML models used in our analysis showed an accuracy rate (F1-score) above 75% in identifying cognitive ability. It was determined that the RF algorithm’s predictive power differed significantly from that of the other models (Table 5).

**Table 4:** 10-Folds cross validation performance measures analysis of the different ML models.

Model	AUC^1^	CA^2^	F1^3^	Precision	Recall	Execution time (s)
RF	0.91	0.87	0.86	0.88	0.87	0.62
ANN	0.85	0.84	0.84	0.84	0.84	20.0
SVM	0.84	0.84	0.83	0.84	0.84	10.2
GB	0.85	0.83	0.82	0.82	0.83	8.1
Decision tree	0.77	0.80	0.81	0.81	0.80	0.03
KNN	0.78	0.77	0.76	0.76	0.77	0.001

1 AUC, area under the curve.

2 CA, correspondence analysis.

3 F1-score, a harmonic mean between precision and recall.

**Table 5:** Model evaluation comparison through Wilcoxon signed rank test.

Pair-wise comparison	*Z*-value	*p*-value
RF-GB	−2.5	0.027
RF-SVM	−2.8	0.002
RF-KNN	−2.8	0.002
RF-DT	−2.8	0.002
RF-ANN	−2.14	0.16
GB-ANN	−2.75	0.004
GB-KNN	−2.8	0.002
GB-SVM	−2.8	0.002
GB-DT	−2.75	0.004
SVM-KNN	−2.8	0.002
SVM-DT	−2.8	0.002
SVM-ANN	−1.43	0.87
KNN-DT	−2.8	0.002
KNN-ANN	−2.8	0.002
DT-ANN	−2.8	0.002

**Figure 1:**
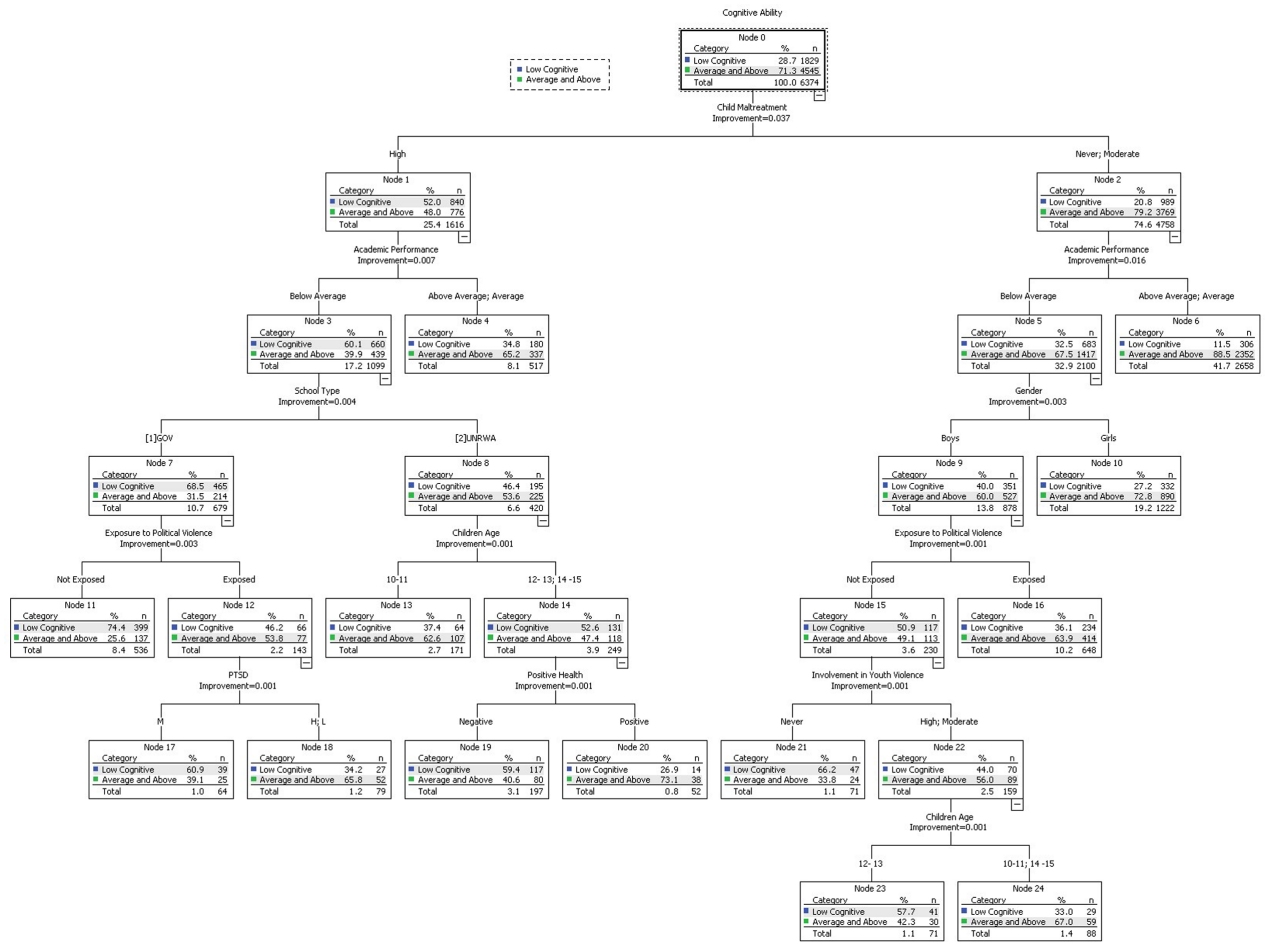
Cognitive ability and the associated factors classification tree.

The CRT analysis results are illustrated in Figure 1 showed that cognitive ability was highly affected by children’s maltreatment, in which the high level of maltreatment had a high percentage of low cognitive scores (52%). Participants who reported experiencing high levels of maltreatment exhibited lower academic performance, which subsequently decreased their cognitive ability. This was evidenced by the fact that 60.1% of participants who had below-average academic scores reported low cognitive ability. Furthermore, the below-average academic performance group was associated with school type and exposure to political violence, in which the public schools had lower cognitive scores, and were more likely to be affected by exposure to political violence. The exposed students in governmental schools were further classified by level of PTSD.

On the other hand, the UNRWA schools were classified by age and positive health perception. Interestingly, the 12–13 and 14–15 age groups were merged into one cluster and reported a high level of low cognitive score (52.6%). Moreover, the negative health perception group reported a higher rate of low cognitive ability (59.4% compared to 26.9% of positive perception).

The other side of the classification tree identified different patterns of association, the maltreatment (never, and moderate) groups were affected by academic performance, whereby the students with below-average academic performance had a higher rate of low cognitive ability (32.5%). Furthermore, the same group was affected by gender, whereby boys reported a higher rate of low cognitive scores than girls. Interestingly, boys were affected by the exposure to political violence.

**Figure 2:**
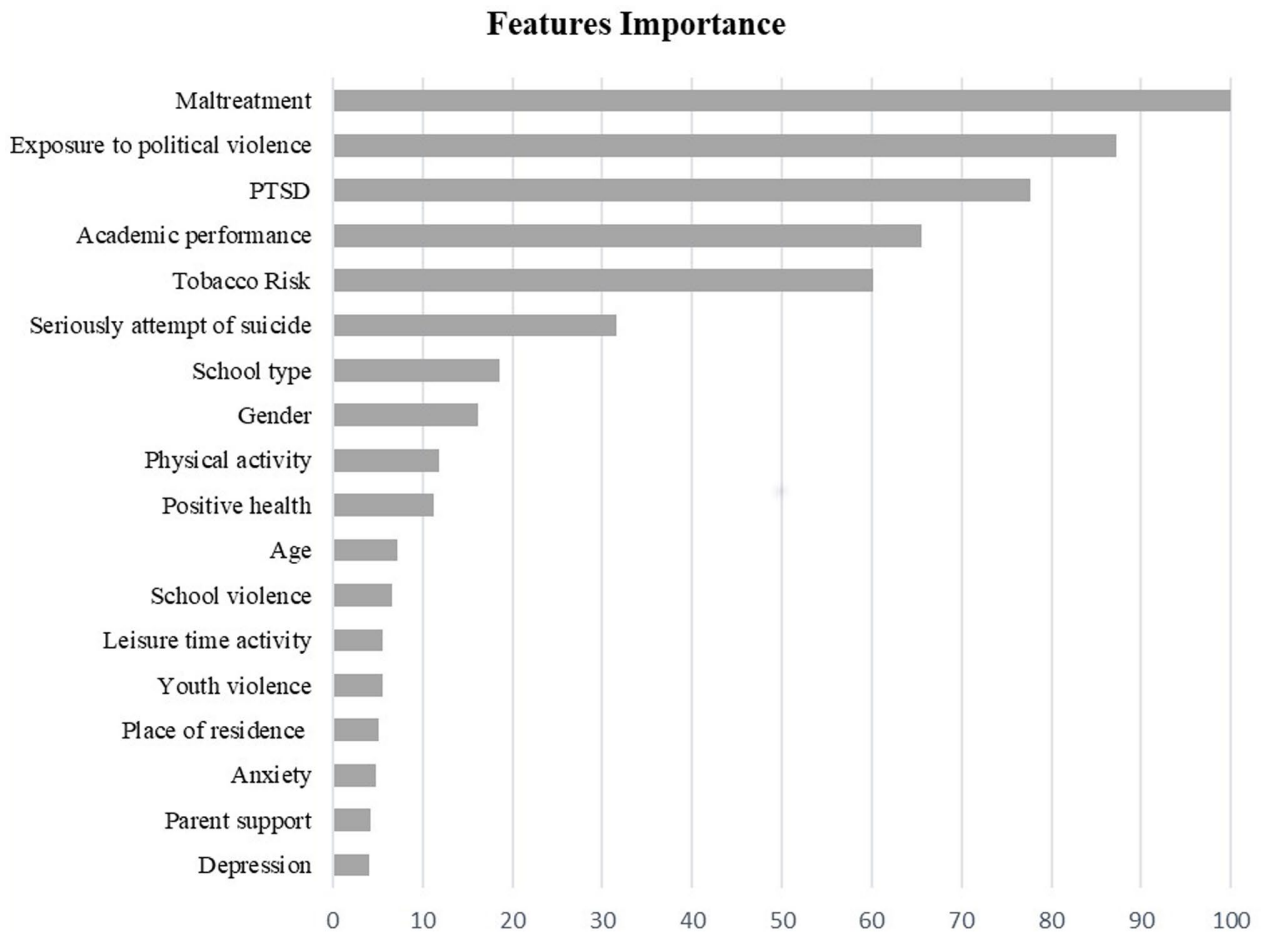
Cognitive ability associated risk factors importance ranking.

Gini Importance analysis was conducted to identify the factors that have the most impact on the likelihood of developing low cognitive ability among schoolchildren. The results of the Gini Importance analysis are illustrated in Figure 2. The findings indicate the relative importance of each risk factor, with the highest-scoring factors being the most significant in identifying cognitive ability. Maltreatment, exposure to political violence, PTSD, academic performance, smoking, suicide attempts, school type, and gender were the most important factors affecting cognitive development among children.

## 4. Discussion

This study utilized a novel approach using machine learning to gain a deeper understanding of the complex relationships between important determinants of cognitive functions (1, 2, 5, 35) that can significantly impact children’s emotional and psychological well-being, as well as their cognitive abilities such as memory, attention, and problem-solving skills (34). The study also examined the impact of severe stress caused by living in a violent environment, which can lead to physiological changes in the brain and body that affect cognitive functioning even after the violence has subsided (3, 41). By using machine learning, the study was able to identify new patterns of associations that traditional statistical models may have missed.

The study evidenced a significant association between cognitive ability scores and gender, place of residence, father’s education, mother’s education, physical activity, leisure time activity, parents’ support, and school type. The findings indicated that the prevalence of low cognitive ability among boys is higher than among girls. Our result is consistent with other research studies that showed girls had better cognitive skills and academic achievement (42, 43). Children living in urban places where more exposed political violence are at higher risk of developing ill mental health than children living in rural areas, which might negatively affect their cognitive development. These findings are consistent with other studies that evidenced the significance of place of residence with cognitive development (13, 14, 34).

Furthermore, the findings indicate that exposure to political violence, PTSD, depression, Behaviours Strengths, and Difficulties scale (emotional symptoms, conduct problems, hyperactivity, peer relationship problems, and prosocial behavior), positive health perception, and serious thoughts of attempting suicide were highly significant with cognitive ability scores. The findings indicated that this effect might be higher among children with low socio-economic status. Our results are consistent with other studies that investigated the effect of ongoing political violence on children’s mental health and cognitive development, in which a strong association between political violence and low academic achievement were found (1, 2, 41). In terms of cognitive ability, research has shown that exposure to political violence can lead to a decline in IQ scores, memory loss, and decreased attention span. This is likely due to the high levels of stress and traumatic experiences associated with such violence, which can disrupt normal brain functioning and lead to long-lasting changes in the brain structure and function (4, 36).

In our study, the CRT classification model revealed different patterns of associations among participants. The study showed that maltreatment was the most important factor related to cognitive development. The CRT model classified maltreatment into two clusters: (1) High levels of maltreatment, and (2) Never or moderate maltreatment. The first cluster (high levels of maltreatment) showed associations with academic performance, school type, and exposure to political violence. This is evidenced by the fact that children with lower academic performance, as well as public-school students had higher levels of association to political violence. In turn, children with higher levels of exposure to political violence showed higher levels of PTSD, which further negatively affected cognitive ability.

The second cluster (moderate or no levels of maltreatment) evidenced a direct association with higher academic performance and gender. Boys’ academic performance is conversely associated to their extent of exposure to political violence, and youth violence, whereby lower exposure to violence among boys is associated with higher academic scores. This can be due to the direct effects of traumatic experiences, exposure to loss and harm, and disruptions to social and personal relationships, especially among boys. These findings are consistent with other studies that indicated a strong association between child abuse, exposure to political violence, and posttraumatic events (2, 5, 35, 36).

The study evidenced that children who experience mental health problems, such as depression and anxiety, may have difficulties with attention and memory, which can affect their academic performance. Furthermore, the study emphasized the fact that the negative impacts of political violence on children’s mental health and cognitive ability can have serious effects, including difficulties with learning, emotional regulation, and overall well-being.

Upon comparing the results of the ML model with those of the logistic regression model, it was found that the ML was able to detect novel patterns of associations between cognitive ability and risk factors. Specifically, the ML model identified maltreatment, PTSD, smoking, and suicide attempts as important factors that might affect the cognitive ability of school children. On the other hand, these factors were not identified as significant in the logistic regression model. The advantage of using the ML model is that it can identify non-linear relationships and interactions between predictor variables that may be missed by traditional statistical models such as logistic regression.

In particular, the ML model identified maltreatment as a significant risk factor for decreased cognitive ability, which is consistent with previous research in this area. However, the model also identified PTSD, smoking, and suicide attempts as additional risk factors that have not been widely studied in the context of cognitive ability in school children. The ability of the ML model to identify novel risk factors underscores the potential usefulness of this approach in identifying previously unknown or overlooked factors that impact cognitive ability. These findings suggest that the ML model may be a valuable tool for future research in this area, as well as for identifying interventions and treatments that may help to mitigate the negative impact of these risk factors on cognitive ability in school children.

The study showed the power of ML tools and algorithms in understanding and addressing cognitive development in a holistic and comprehensive approach that considers the complex interplay between mental health, cognitive ability, and exposure to political violence. Moreover, the utilization of advanced algorithms and tools, researchers can more accurately identify patterns and trends in complex datasets, which can help them to better understand the relationship between mental health and cognitive ability in this vulnerable population. It is important to note that the use of ML in identifying mental health and cognitive ability is still in its early stages, and there is a need for further research to validate and improve the balanced accuracy of these models.

### 4.1. Strengths and limitations

The relationship between cognitive development and mental health has been investigated by several research studies evidencing a strong interrelation between the two factors. To the best of our knowledge, the current study is considered the first of its kind that deploys ML techniques in assessing the relationship between socio-economic, mental health social factors, lifestyle and exposure to political violence, and cognitive ability among children living within an ongoing politically violent environment. The use of ML provides an in-depth understanding of the nature of these associations and identified new patterns of associations. The studied ML models are less dependent on the linear relationship between risk factors, which could provide a more precise and accurate association.

The ML model was developed to detect novel patterns of associations and identified maltreatment, PTSD, smoking, and suicide attempts as important risk factors affecting the cognitive ability of school children. On the other hand, these factors were not identified as significant in the logistic regression model. Our results highlight the potential usefulness of ML in identifying non-linear relationships and interactions between predictor variables that may be missed by traditional statistical models such as logistic regression. This, in turn, will enhance the development of precise and efficient intervention programs that improve children’s growth and cognitive skills development in Palestine and other politically violent environments. Thus, this research study not only introduces the methodology of ML techniques in identifying cognitive abilities but also provides decision-makers with the power of ML in the early diagnosis of schoolchildren’s cognitive skills.

Nonetheless, the study is limited by several factors, such as the target region was selected from the Palestinian community only, the levels of political violence, and the participants’ age group that does not include children under 12 years and adults >18 years. However, future research will benefit from this study by adding other risk factors. Additional studies can be conducted related to other external factors, such as forced displacement, house demolitions, poverty, family social problems, and mobility limitations. The integration of the above variables would provide an in-depth understanding of cognitive abilities development among schoolchildren in the Palestinian context. The presence of these variables would further enhance the accuracy of the ML models’ identification for cognitive abilities.

## 5. Conclusion

The findings of this study offer important insights into the complex interplay between various risk factors and cognitive development in children in conflict zones. The use of ML techniques allowed for the identification of factors associated with cognitive ability and mental health risks in a novel and data-driven approach, highlighting significant risk factors such as exposure to political violence, maltreatment, severe mental health disorders, and below-average academic performance.

This study contributes to the growing literature on the effects of adverse childhood experiences and underscores the need for policymakers, practitioners, and researchers to address the negative impact of political violence on children’s well-being. Additionally, the study’s identification of gender differences in the relationship between academic performance and cognitive ability emphasizes the need for targeted interventions to support boys exposed to political violence.

Practically, this study’s findings can inform evidence-based strategies for preventing and mitigating the detrimental effects of political violence on individuals and communities. Policymakers, practitioners, and researchers can utilize the insights gained from this study to design interventions aimed at supporting and treating children impacted by violence and promoting safer environments for their growth and development. Moreover, the performance of the developed ML model (precision and recall >85%) is encouraging and can guide future research in the development of a tool to support the identification of children at risk of having low cognitive abilities.

## Data availability statement

The original contributions presented in the study are included in the article/supplementary material, further inquiries can be directed to the corresponding author.

## Ethics statement

The studies involving human participants were reviewed and approved by Al-Quds University IRB. Written informed consent to participate in this study was provided by the participants’ legal guardian/next of kin.

## Author contributions

RQ, ZA, SH, and DA: conceptualization and methodology. RQ: formal analysis, validation, and writing original draft preparation. DA and SV: editing and review. RQ, NA, and RA: data curation and data pre-processing. All authors contributed to the article and approved the submitted version.

## Funding

The work included in this study is part of the wider project Determinants of Cognitive Development in Deprived Environments: evidence from the West Bank funded by the German Research Foundation (DFG) under Grant Number JU 2769/2.

## Conflict of interest

The authors declare that the research was conducted in the absence of any commercial or financial relationships that could be construed as a potential conflict of interest.

## Publisher’s note

All claims expressed in this article are solely those of the authors and do not necessarily represent those of their affiliated organizations, or those of the publisher, the editors and the reviewers. Any product that may be evaluated in this article, or claim that may be made by its manufacturer, is not guaranteed or endorsed by the publisher.
